# Temperature-Dependent Infrared Refractive Index of Polymers from a Calibrated Attenuated Total Reflection Infrared Measurement

**DOI:** 10.1177/00037028221094598

**Published:** 2022-05-25

**Authors:** Md S. Azam, Malcolm D. Ranson, Dennis K. Hore

**Affiliations:** 1 Department of Chemistry, 8205 University of Victoria, Victoria, BC, Canada; 2 Department of Computer Science, 8205 University of Victoria, Victoria, BC, Canada

**Keywords:** Attenuated total reflection infrared spectroscopy, ATR-IR, polarization, susceptibility, complex refractive index, temperature-dependence, polymers

## Abstract

We demonstrate a straightforward method by which a commonly available reference sample such as water can be used to calibrate an attenuated total internal reflection infrared absorbance measurement in order to account for the polarization of the beam incident on the internal reflecting element, and the spread of angles about the nominal angle of incidence. This enables quantitative comparison of attenuated total reflection-derived absorbance data with spectra calculated from optical constants. We then apply this calibration to the measurement of temperature-dependent absorption spectra of a polydimethylsiloxane sample. We illustrate that the extracted optical constants scale with the temperature-dependent changes in the polymer density better than the raw absorbance values on vibrational resonance.

## Graphical abstract

**Graphical Abstract:**
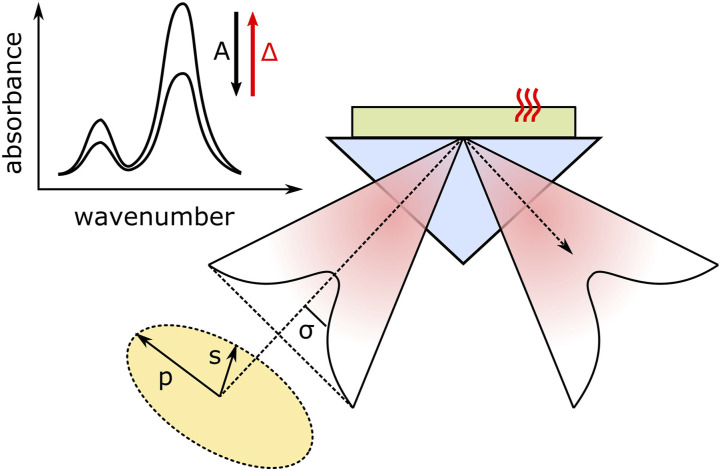

## Introduction

The optical constants of materials are the fundamental properties necessary for a wide range of applications in chemistry, physics, and materials science and engineering. This also encompasses many situations in which the individual materials may not be the target of interest, but the optical constants of all components of a more complex system or device are needed to understand its function. There are many methods that can be employed for such characterization, including refractometry,^1,2^ reflectometry,^3–6^ and ellipsometry.^7–11^ However, these measurements continue to be a challenge in the mid-infrared primarily as a result of low-intensity uncollimated incoherent light sources and more limited options for broadband polarization control. And yet the infrared spectral region contains rich chemical information on a sub-molecular scale on account of distinct vibrational resonances of localized chemical functional groups. Another area of importance is the interconversion of different spectral measurements, such as those in transmission- and reflection-based geometries. This is particularly valuable in the utilization of infrared spectral libraries when decomposing mixtures into constituent components or fitting absorption lineshapes.^12–15^

Although some of these shortcomings are poised to be overcome with the advent of mid-infrared quantum cascade lasers^16,17^ coupled with microbolometer arrays,^18,19^ Fourier transform infrared (FT-IR) instruments continue to be the workhorse for laboratory, industrial, and field work. In recent years, attenuated total internal reflection infrared (ATR-IR) spectroscopy has largely taken over, and is either a common accessory or the standard configuration of many FT-IR instruments.^20–22^ This is primarily due to the convenience of being able to sample materials that are large, irregularly-shaped, and thick/opaque without any preparation, making use of the limited evanescent wave penetration to overcome the path length restriction of transmission measurements. Although it is useful to consider the evanescent wave penetration depth when the angle of incidence exceeds the critical angle,^23^ strictly speaking, the field is purely evanescent on the rarer side of the internal reflecting element (IRE) only when the rarer medium is transparent.^20^ In ATR-IR, the goal is to study absorbing materials; the imaginary part of the refractive index enables energy to be transferred into the material thereby decreasing the reflectance in a manner that is eventually expressed as absorbance. Several approaches make use of this phenomenon and the ATR sensitivity to both the real and imaginary components of the refractive index to determine the optical constants of materials,^24,25^ including methods that utilize unpolarized^26^ and polarized light,^27^ and the simultaneous use of two orthogonal beam polarizations.^28^ The general approach has been to estimate the imaginary part of the index (often through an assumption that it is proportional to the ATR absorbance for weak bands) and then perform an iterative fitting to arrive at self-consistent solutions to the dispersion of the complex refractive index that satisfy Kramers–Kronig relations. This approach has also been extended to the characterization of aniostropic materials.^29,30^

For IR transmission spectra, particularly for samples diluted in an IR-transparent matrix such as KBr, the imaginary part of the refractive index is readily obtained (albeit with altered local field effects) and can be related to the absorption coefficient.^29,31^ ATR-IR spectra, however, trade-off sample measurement convenience for some additional complexity in the analysis. This is due primarily to the fact that both the real and imaginary components of the refractive index contribute to the ATR-IR line shape.^32^ One method commonly employed in the quantitative analysis of ATR-IR spectra is to consider the frequency-dependent evanescent wave penetration depth, and then use this as the effective sample thickness in a Beer-Lambert model.^33^ The challenge is that, such an approach still requires knowledge of the real part of the refractive index. A further complication that impedes quantitative analysis of ATR-IR spectra is the polarization of the input beam. This is largely irrelevant in a transmission measurement at normal incidence. However, since reflection measurements are performed at oblique incidence, the relative contributions of the s- and p-polarized reflectance depends on the input beam polarization.^32^ Even when using unpolarized blackbody sources, the many reflections inside the spectrometer and inside the ATR-IR accessory often impart a significant polarization to the beam incident on the sample. This can be countered by the use of polarizers to control the input polarization state, but at the expense of further reducing the incident beam intensity. Finally, the reflectance in the vicinity of the critical angle is very sensitive to the angle of incidence. The beam approaching the sample compartment in an FT-IR instrument can have a diameter of up to several centimeters. This then needs to be tightly focused to accommodate an IRE with an area of only a few square millimeters. Previous studies have made use of this angle spread in order to vary the evanescent wave penetration depth, particularly in ATR-IR imaging experiments.^34–37^

In this account, we describe a straightforward manner for calibrating the response of any given ATR-IR setup using a commonly-available reference material in order to characterize the input polarization state and beam divergence. We then use these parameters to measure and model the ATR-IR spectra of a polymer as a function of temperature. We illustrate that, although the absorbance decreases with increasing temperature as expected when the density decreases, quantitative scaling is observed only for the imaginary part of the refractive index itself, or the extracted oscillator strength.

## Methods

Polydimethylsiloxane (PDMS) films were prepared from a Sylgard-184 kit (Dow Corning) on glass microscope slides (Fisherbrand Adhesion Slides, Thermo Fisher) that were cleaned with acetone and oven dried (30 min at 100 °C) prior to use. PDMS part A (base) and part B (curing agent) were well-mixed in a 10:1 ratio by stirring for 5 min. The mixture was then degassed by placing it in a vacuum desiccator for 30 min. The glass slides were then spin-coated (Specialty Coating Systems, Inc) with the as-prepared PDMS mixture at 1200 rpm for 1 min. The samples were then cured at 85 °C for 4 h. This procedure resulted in films with a nominal thickness of 10 *μ*m.^38^

After curing, the samples were placed PDMS side down onto a single-bounce diamond 45° internal reflecting element installed in an FT-IR (Bruker Vertex 70) fitted with a KBr beamsplitter and DTGS detector. The ATR accessory (Pike GladiATR) enabled temperature control from ambient to 200 °C. Each spectrum was collected as a co-addition of 64 interferograms with a 2.5 kHz scanner velocity. A Mertz phase correction was applied and a Blackman-Harris three-term apodization function was used. Background measurements (of the IRE–air interface) were taken at each temperature in order to remove temperature-dependent artifacts.^39^ Such spectral processing, that requires reading background and sample spectra from different files, was facilitated by the OpusFC python library.^40^

For the instrument calibration, 18.2 MΩ⋅cm deionized water and anhydrous ethanol (Sigma Aldrich) were used, together with established optical constants from the literature.^41,42^

## Basic Concepts and Film Thickness Considerations

In contrast to the absorptance, the quantity absorbance lacks a consistent definition, as it is obtained with respect to some kind of reference spectrum. In general the absorbance is obtained from

(1) $$A=-\log_{10}\left(\frac{R_{\text{sample}}}{R_{\text{ref}}}\right)=-\log_{10}\left(\frac{{\left|r_{\text{sample}}\right|}^{2}}{{\left|r_{\text{ref}}\right|}^{2}}\right)$$

where *R* is the reflectance, the ratio of the intensity of the reflected light *I* to that of the incident light *I*_0_. The choice of reference depends on the intent of the experiment. For example, for aqueous samples, water is often chosen. If we consider the ratio of light reflected with the sample on the IRE compared to the reference state of no sample (IRE–air interface),

(2) $$\frac{R_{\text{sample}}}{R_{\text{ref}}}=\frac{I_{\text{sample}}/I_{0}}{I_{\text{ref}}/I_{0}}=\frac{I_{\text{sample}}}{I_{\text{ref}}}$$

we can see that a measurement of the incident light intensity *I*_0_ is not required. However, as Eq. 1 involves the Fresnel reflection coefficients *r*, knowledge of the incident angle and beam polarization is required. If we consider light polarized with the electric field parallel (p) and perpendicular (s) to the plane of incidence, the Fresnel coefficients are given by

(3a) $$r_{p}=\frac{n_{1}\cos\theta_{2}-{\tilde{n}}_{2}\cos\theta_{1}}{n_{1}\cos\theta_{2}+{\tilde{n}}_{2}\cos\theta_{1}}$$

(3b) $$r_{s}=\frac{n_{1}\cos\theta_{1}-{\tilde{n}}_{2}\cos\theta_{2}}{n_{1}\cos\theta_{1}+{\tilde{n}}_{2}\cos\theta_{2}}$$

where *n*_1_ is the refractive index of the incident material (the IRE) in this case and ${\tilde{n}}_{2}$ is the complex refractive index of the sample when measuring *I*_sample_ and the (real) refractive index of air when measuring *I*_ref_. Here, we use the notation for the complex index $\tilde{n}=n+ik$ . Since a good IRE is essentially transparent, ${\tilde{n}}_{1}\approx n_{1}$ . For the reference (air) measurements, we assume ${\tilde{n}}_{2}=n_{2}=1$ . In the above expression, θ_1_ is the incident angle at the IRE–sample or IRE–air interface, and θ_2_ is the refracted angle.

When performing measurements on thin films in the typical range of thickness obtained by spin coating polymers (50 nm–10 μm) thin film interference effects need to be considered. Two approaches are possible. One is to explicitly model the interference using precise knowledge of the film thickness (as obtained by profilometry or atomic force microscopy). This is straightforward, and many approaches have been described in the literature.^43,44^ A particularly compact route is to use a transfer matrix in the form of

(4) $$M=\left[\begin{array}{ll} \cos\beta_{2} & -\frac{\text{i}}{p_{2}}\sin\beta_{2}\\ -\text{i}p_{2}\sin\beta_{2} & \cos\beta_{2} \end{array}\right]$$

where $p_{2}^{s}={\tilde{n}}_{2}\cos\theta_{2}$ for s-polarized light, $p_{2}^{p}=p_{2}^{s}/{\tilde{n}}_{2}^{2}$ for p-polarized light,^45,46^ and θ_2_ is the refracted angle. The quantity β_2_ = 2π*dp*_2_/*λ* incorporates the phase difference in propagation through the film. This enables the Fresnel reflection coefficients introduced in Eqs. 3a and 3b to be replaced with

(5) $$r=\frac{p_{1}\left(M_{11}+p_{3}M_{12}\right)-\left(M_{21}+p_{3}M_{22}\right)}{p_{1}\left(M_{11}+p_{3}M_{12}\right)+\left(M_{21}+p_{3}M_{22}\right)}$$

where *p*_1_ and *p*_3_ are defined in an analogous manner to *p*_2_ as described above. The reflectance is then obtained from *R* = |*r*|^2^ in the usual way and the absorbance can be calculated when the reflectance of the sample and reference are compared.

**Figure 1.**
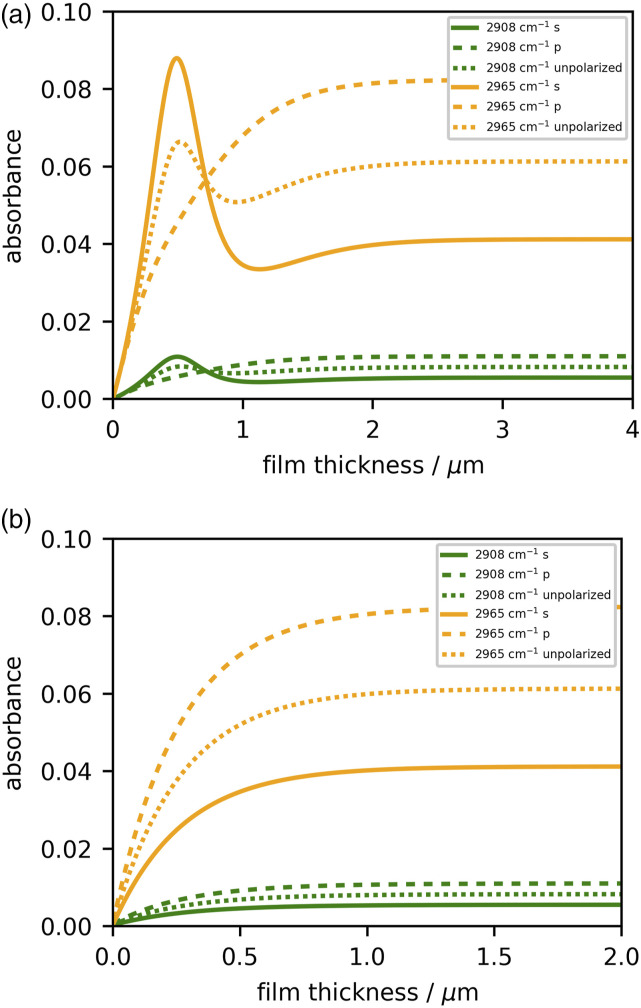
Absorbance as a function of film thickness for a (a) diamond–PDMS–air and (b) diamond–PDMS–glass system.

Another option is to prepare films that are sufficiently thick or thin, thus simplifying the analysis. Fig. 1 plots the expected absorbance calculated using the transfer matrix described above with literature values of ${\tilde{n}}_{2}$ and a nominal angle of incidence of θ_1_ = 45°; the refracted angle θ_2_ is determined from Snell’s law. Even though the literature refractive index data may not exactly match our PDMS sample, a few conclusions can be made. In the case of the free-standing films placed directly on the IRE (IRE_1_–film_2_–air_3_, with ${\tilde{n}}_{3}=n_{3}$ = 1), Fig. 1a indicates that interference effects can be ignored when the film thickness exceeds approximately 2 *μ*m. In our case, since we work with thinner samples, the films are first spin-coated onto glass substrates (IRE_1_–film_2_–glass_3_–air_4_, with glass ${\tilde{n}}_{3}$ data from the literature^47^), and so Fig. 1b indicates that for PDMS thicknesses greater than 1 *μ*m, such modeling is not necessary. The other conclusion from this modeling is that, for sufficiently thin films (less than about 50 nm) a simpler model of absorbance may be employed, but we will not elaborate on that point here.

We also note the significant difference in the behavior of s- and p-polarized beams. If it has been verified that the polarization incident on the IRE is random (seldom the case), the unpolarized absorbance can be determined by using $R_{\text{unpolarized}}=1/2\left(R_{s}+R_{p}\right)$ in place of *R*_sample_ and *R*_ref_ in Eq. 1.

## Results and Discussion

### Determination of ATR-IR Experimental Parameters

**Figure 2.**
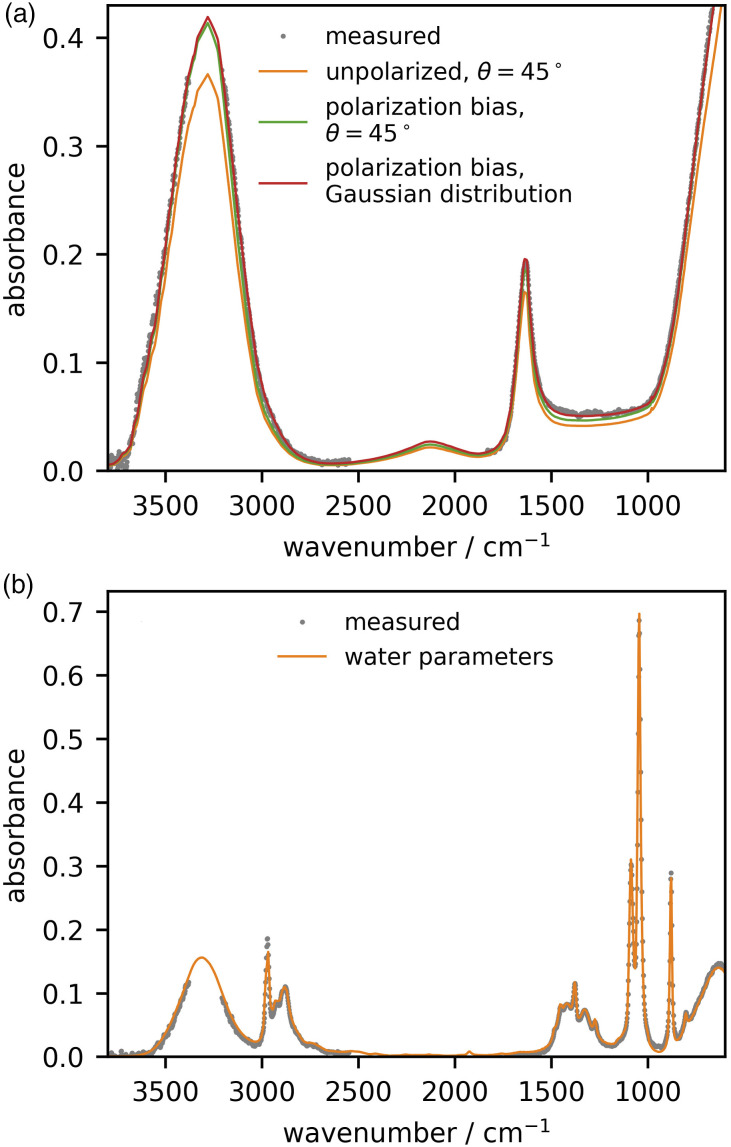
(a) Determination of the instrument parameters (input polarization, and spread of beam angles) using literature *n* and *k* values for water and our measured absorbance spectrum. (b) Application of these values, comparing our measured with the predicted absorbance spectrum of ethanol. In both cases, experimental data points in the atmospheric water bending and stretching modes have been removed for the data fitting so that noise in the absorbance does not influence the parameterization.

Figure. 2a provides a comparison between the unpolarized absorbance spectrum predicted from Eq. 1 using literature complex refractive index for water (orange trace) and our measured spectrum (gray dots). To facilitate subsequent fitting, we have omitted data in the 3300 cm^−1^ and 2350 cm^−1^ regions on account of the noise due to background correction artifacts resulting from atmospheric water vapor and carbon dioxide.

Although the spectral features are similar, the quantitative agreement is poor for two reasons. The first is that it is an unreasonable assumption that the IR beam is randomly polarized, on account of the many reflection that occur inside the FT-IR and within the ATR accessory. If we define the fraction of the incident beam that is s-polarized as *f*, then Eq. 1 should be adapted to consider

(6) $$R=fR_{s}+\left(1-f\right)R_{p}$$

Using the literature values for *n*_2_ and *k*_2_ for water, we fit our experimental data to determine the best value for *f* to minimize the residual, resulting in *f* = 0.32 (green trace in Fig. 2a). One can see that the agreement has vastly improved, but this should be a quantitative comparison. We next consider that, due to focusing of the IR beam onto the sample, there is a distribution of angles about the nominal θ_1_ = 45°.^48,49^ We consider a Gaussian distribution of angles

(7) $$s\left(\theta \right)=c_{N}\exp\left[-\frac{{\left(\theta -\theta_{1}\right)}^{2}}{2\sigma^{2}}\right]$$

with a full width at the half maximum value of σ and the normalization constant is defined as

(8) $$c_{N}={\left[\int_{0}^{\pi }\exp\left[-\frac{{\left(\theta -\theta_{1}\right)}^{2}}{2\sigma^{2}}\right]\sin\theta \text{d}\theta \right]}^{-1}$$

Fitting to this two-parameter model returned *f* = 0.33 and σ = 4.7°, and resulted in a quantitative agreement with the experimental data (Fig. 2a, red trace). One notices that the improvement offered by the spread of beam angles is minor in some parts of the spectrum, but offers noticeable improvements in others such as the 3000 cm^−1^ and 1200 cm^−1^ regions.

In order to demonstrate the applicability of these parameters determined for our instrument, we measure the IR spectrum of another liquid, ethanol, that has substantially different ${\tilde{n}}_{2}$ in the mid-IR, and compare with the absorbance we calculate from literature index data in the same spectral region, using the same values of *f* and σ that we have previously determined for water. The results presented in Fig. 2b show very good quantitative agreement. This method is quick to perform on any instrument. We have used water for the calibration: The ideal reference sample is a liquid, since good contact with the IRE is ensured, and one for which the literature *n*_2_ and *k*_2_ values have been determined simultaneously and independently through a robust method, such as spectroscopic ellipsometry. This is preferable to data sets where *n*_2_ has been determined from experimental *k*_2_ from fitting or Kramers–Kronig approaches, as there are additional approximations inherent in such calculations. It is also desirable that *k* be sufficiently large across a wide range of wavelengths.

### Determination of PDMS Complex Refractive Index in the C–H Stretching Region

**Figure 3.**
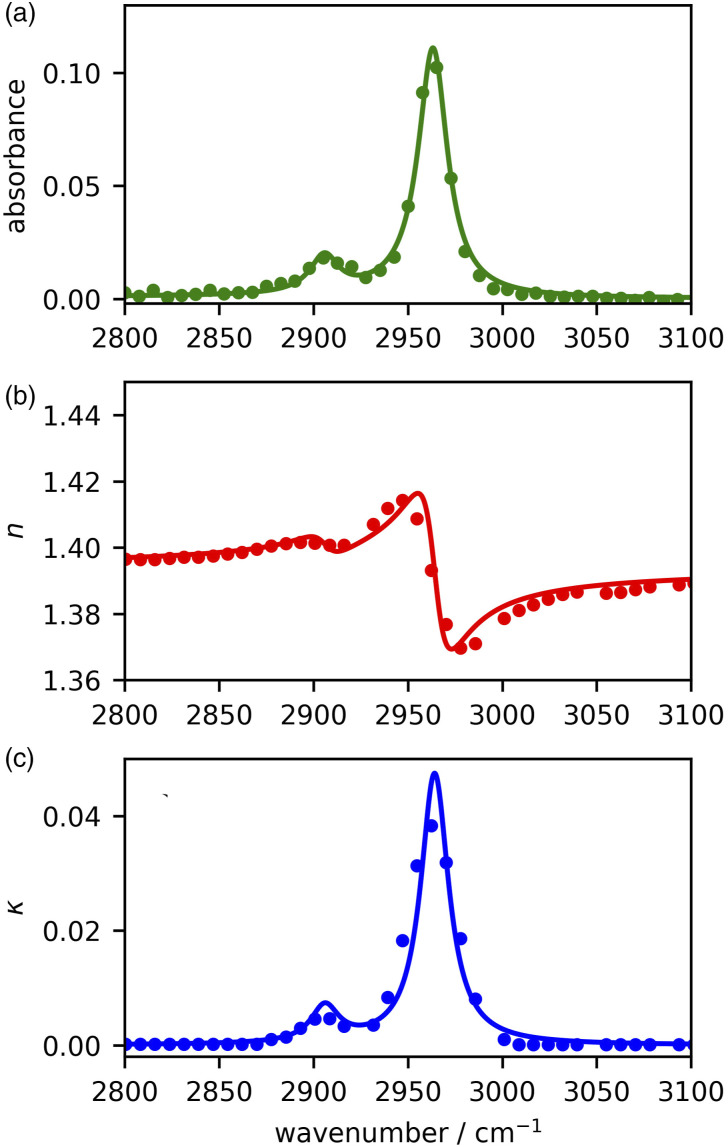
(a) Measured (points) and fit value (lines) of the PDMS absorbance spectrum using the Lorentz oscillator model defined in Eq. 9. This results in the (b) real and (c) imaginary components of refractive index, plot in lines based on the model fit parameters. For comparison, the literature *n* and *k* values are superimposed in points (panels b and c), although these are not necessary from the same type of sample.

Figure. 3a shows the absorbance measured from our PDMS film at room temperature (experimental data plotted in points). The high frequency region consists of two modes, the methyl symmetric stretch near 2910 cm^−1^ and the methyl antisymmetric stretching near 2960 cm^−1^.

Since this region of the spectrum, of interest to us for related surface studies, is so simple, we can easily use a two-oscillator model for the material susceptibility function *χ* that relates the dipole moment per unit volume *P* to the electric portion *E* of the infrared electromagnetic field through *P* = *ɛ*_0_*χE*. We use a Lorentz model^50^

(9) $$\chi \left(\omega \right)=A_{\text{NR}}+\sum_{j=1}^{2}\frac{A_{j}}{\omega_{j}-\omega -i\Gamma_{j}}$$

where *A*_NR_ is a vibrationally non-resonant contribution to the susceptibility, $i=\sqrt{-1}$ , *A*_1_ and *A*_2_ are the amplitudes, *ω*_1_ and *ω*_2_ the resonance frequencies, and Γ_1_ and Γ_2_ the linewidths (HWHM) of the methyl symmetric and antisymmetric stretching modes. The frequency-dependent complex refractive index of PDMS in this spectral region is then given by

(10) $${\tilde{n}}_{2}\left(\omega \right)=n_{2}\left(\omega \right)+ik_{2}\left(\omega \right)=\sqrt{1+\chi \left(\omega \right)}$$

**Figure 4.**
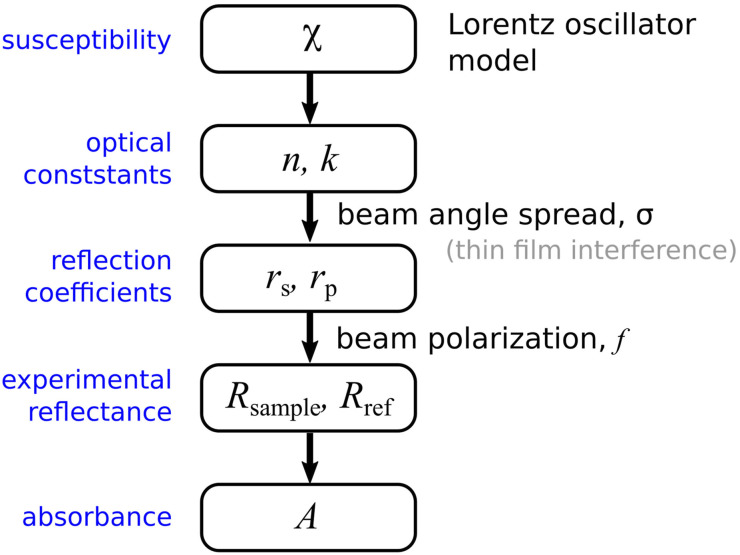
A Lorentz model (Eq. 9) describing each of the resonant modes is constructed and used to determine the complex refractive index via Eq. 10. The distribution of beam angles (Eq. 7) is then used to evaluate the reflection coefficients, including possible thin film interference if appropriate. Finally, the reflectance of the sample and reference (air) is determined using the polarization of the beam (*f* parameter) to ultimately arrive at the absorbance (Eq. 1).

The seven parameters in Eq. 9 then enable the PDMS refractive index to be determined via Eq. 10. The fitting works by comparing with the experimental absorbance spectrum through knowledge of the previously characterized instrument parameters *f* and σ. A scheme of the overall procedure is provided in Fig. 4. Historical approaches to determining the optical constants from ATR-IR measurements typically use an iterative procedure to fine tune the optical constants using a Kramers–Kronig constraint. In our approach, such a constraint is not required as we start with a Lorentz model expression that inherently satisfies the Kramers–Kronig condition.

A truncated Newton’s algorithm was used to find best fit values of the Lorentz model parameters and resulted in *A*_NR_ = 0.943, *A*_1_ = 0.159, *A*_2_ = 1.19, ω_1_ = 2906 cm^−1^, ω_2_ = 2964 cm^−1^, Γ_1_ = Γ_2_ = 9 cm^−1^ to produce the line plotted in Fig. 3a. Once these seven material parameters have been determined, Eqs. 9 and 10 can be used to plot *n*_2_ and *k*_2_ (lines in Figs. 3b and 3c). Although our PDMS sample is not necessarily the same as that for which optical constants are reported in the literature (there is the possibility of minor differences in cross linking agents and ratios of components), published values of *n*_2_ and *k*_2_ are plotted (points in Figs. 3b and 3c) for comparison. Even though this comparison may not be fully quantitative, we can see that, after the instrument response is calibrated, this method can be used to determine the refractive index of such samples.

Although the samples we have studied are sufficiently thick to avoid the need of explicit thin film interference modeling, an intriguing possibility for the approach we have outlined is that thin samples are handled just as easily. The basis of our model is the connection between optical constants (obtained from the dispersion of the susceptibility) and the reflectance using the polarization bias and spread of beam angles. Therefore the reflectance calculated via the transfer matrix approach would also incorporate *f* and σ in a straightforward way, if needed, as shown in the second step in Fig. 4.

### Temperature-Dependence of the PDMS Infrared Optical Properties

**Figure 5.**
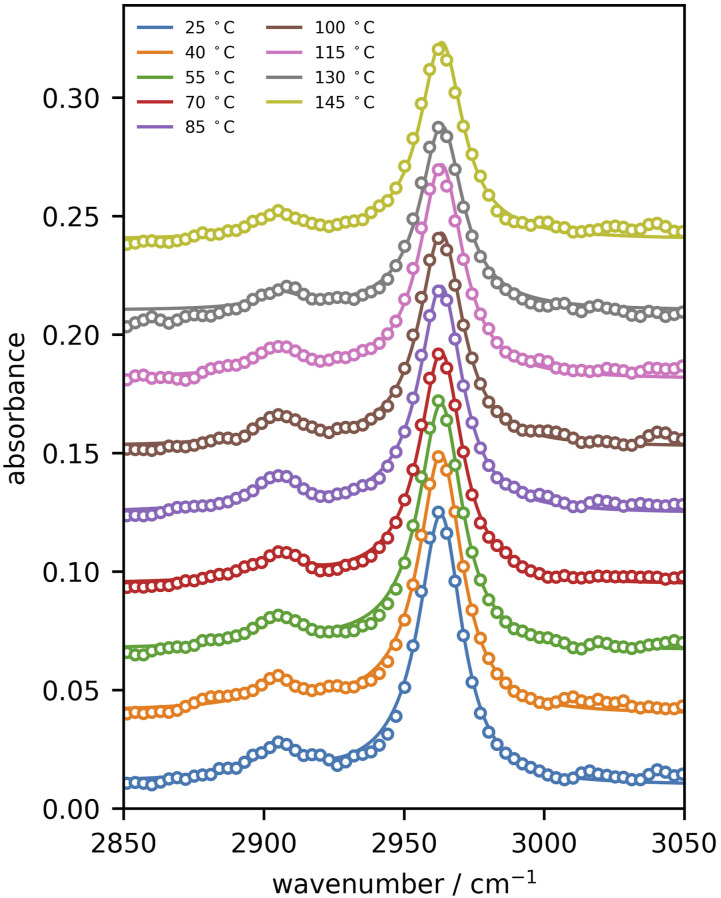
Absorbance spectra obtained upon heating the thick PDMS film, with experimental data plotted in points, and a fit to Eq. 9 plotted with lines.

Using the same PDMS sample, spectra were recorded as a function of temperature in the range 25–145°C in steps of 15 °C and displayed in Fig. 5 (points), together with a fit to Eq. 9 (lines). An investigation of the temperature-dependence of the methyl symmetric and antisymmetric resonance frequency revealed tightly clustered values with no temperature trend. As a result, Γ_1_ and Γ_2_ were fixed to the mean values of 9 cm^−1^.

**Figure 6.**
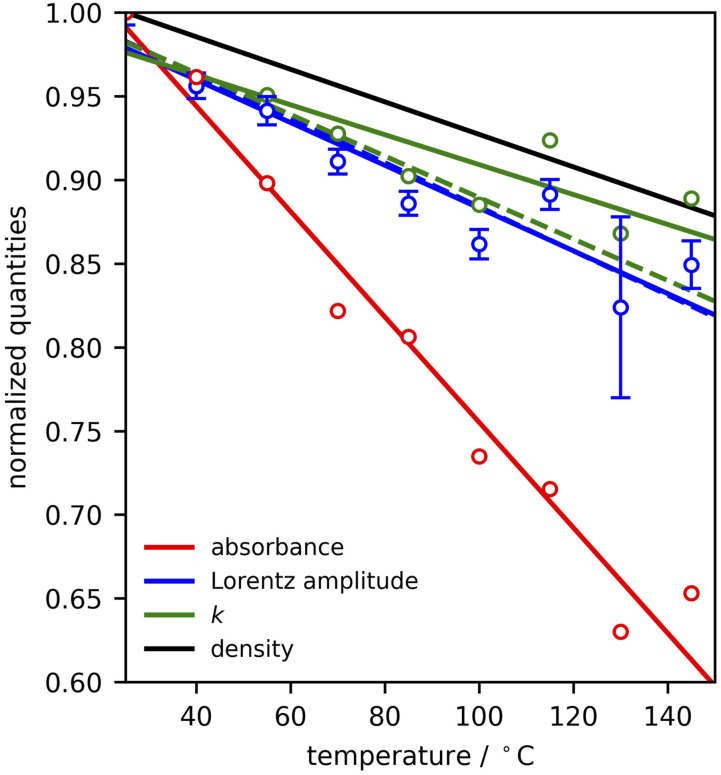
Absorbance values (red), fit amplitudes of the 2964 cm^−1^ mode (blue) with error bars based on the covariance of the fitting parameters, modeled imaginary part *k* of the refractive index (green), all normalized with respect to their values at 25°C. Corresponding solid lines are linear fits to the normalized quantities. The density (normalized to its value at 25°C is plotted in black.^52^ For comparison, values of *A* and *k* determined without considering the spread of beam angles are plotted with dashed lines of the same color.

We now plot the temperature-dependence of the absorbance at 2964 cm^−1^ in Fig. 6 with red circles. Superimposed on the data are the Lorentz amplitudes (blue circles) with error bars determined based on the covariance of the fitting parameters in Eq. 9, and the imaginary part of the refractive index (green points) obtained from Eq. 10. To illustrate the trend in these values with temperature, we have performed linear fits, displayed with solid lines of the corresponding colors. We then compare these trends with the temperature-dependence of the density,^51,52^ displayed with the black line. In order to highlight the degree to which these features change with temperature, Fig. 6 plots all quantities normalized to their value at 25°C.

The oscillator strength, determined by a combination of the transition dipole moment and the local field effects in the condensed medium, is represented by the amplitude *A* in Eq. 9. Furthermore, for weak oscillators, as is typical of organic materials in the mid-infrared, the shape of the susceptibility function should closely match the absorption coefficient. However, ATR-IR data generally does not allow the optical constants to be separated in the same way that *k* can be obtained from a transmission measurement using an appropriate reference sample. The consequence is that both *n* and *k* contribute to the absorbance. It is generally understood that, when there is no significant change in phase or molecular interactions, optical properties scale with density.^53^ Our results indicate that, when *k* is extracted, this is indeed the case, owing to the close agreement in the ≈10% drop in both quantities over the studied temperature range. We also see that the susceptibility on resonance (blue trace in Fig. 6), follows roughly the same trend as one would expect. However, as a result of the nature of an ATR-IR experiment the absorbance itself, although it follows the same downward trend with the increase of temperature, is not as good a proxy for the density change, as it drops by nearly 60% in this interval. The large difference in the temperature trends of the absorbance and *k* is due to a combination of the temperature-dependent non-resonant contribution to the susceptibility and the spread of beam angles. When either the non-resonant/resonant ratio or the spread of angles decreases, the red and blue/green lines are closer together. It is also noteworthy that, under the present conditions, the ≈5° spread results in the largest deviation between these quantities. As σ increases, the agreement improves. It also is interesting to compare the results of this analysis to what would be obtained by neglecting the spread of beam angles. The resulting amplitudes and extracted imaginary part of the refractive index are indicated by dashed blue and dashed green lines, respectively, in Fig. 6. Here, we see that the amplitude and *k* are in closer agreement, as one would expect for a relatively isolated mode on resonance. However, the trend in the optical constant *k* does not agree as well with the density trend as when the spread of beam angles is considered.

One potential limitation of this method is that it requires spectral fitting and the inherent lineshape modeling. In the example we presented, this was straightforward as we had only two bands in a small wavenumber region. When the spectral region of interest widens, it becomes increasingly likely that one encounters many vibrational modes including ones that are overlapping. The proposed approach should still work, however, as it is routine to fit congested spectra in mid-infrared spectroscopic ellipsometry with a large number of modes.^54^

## Conclusion

We have illustrated a straightforward way to perform an ATR-IR experiment with an uncontrolled input polarization state and uncharacterized beam focusing parameters, and still make a connection to the optical constants of materials. The instrument characterization is carried out using a simple optical model and a commonly-available reference sample. As an application of this method, we have collected ATR-IR spectra of polydimethylsiloxane as a function of temperature and found that the absorption coefficient, when extracted from a fit to the data, matches the density change as expected. This type of analysis is enabled by restoring the relationship between the ATR-IR absorbance and the material optical constants.
